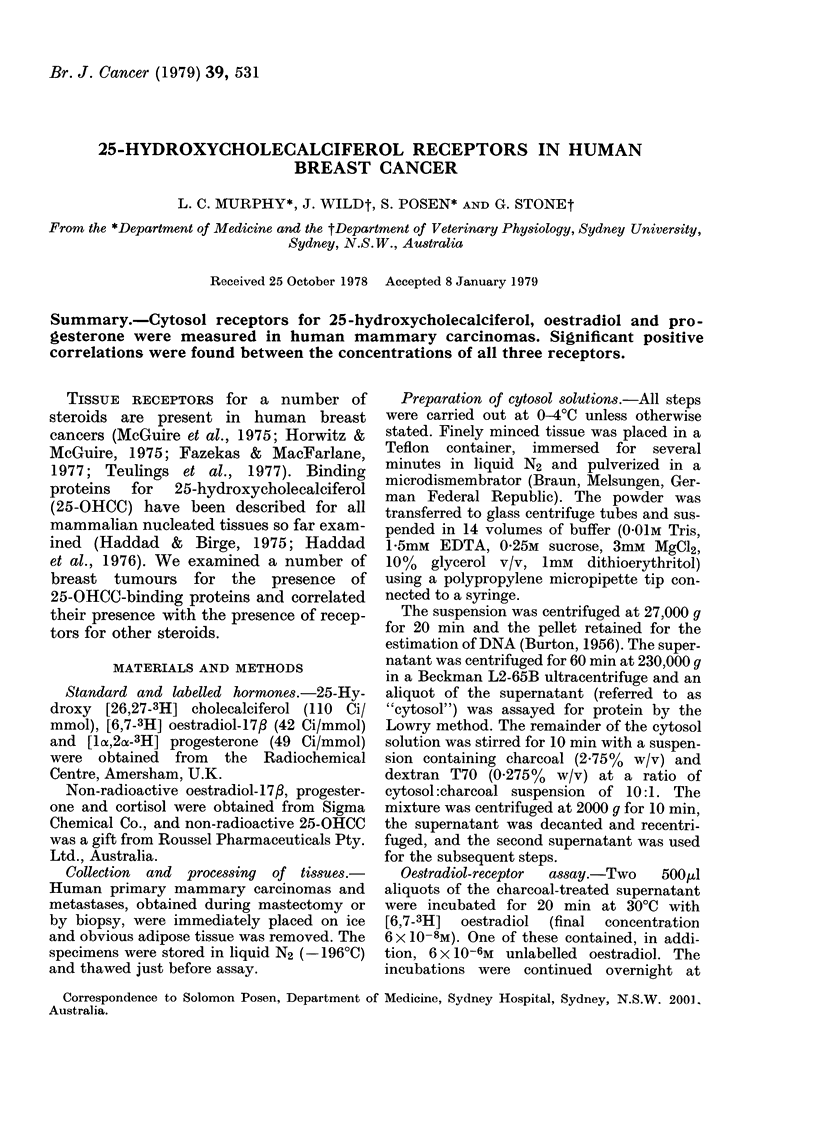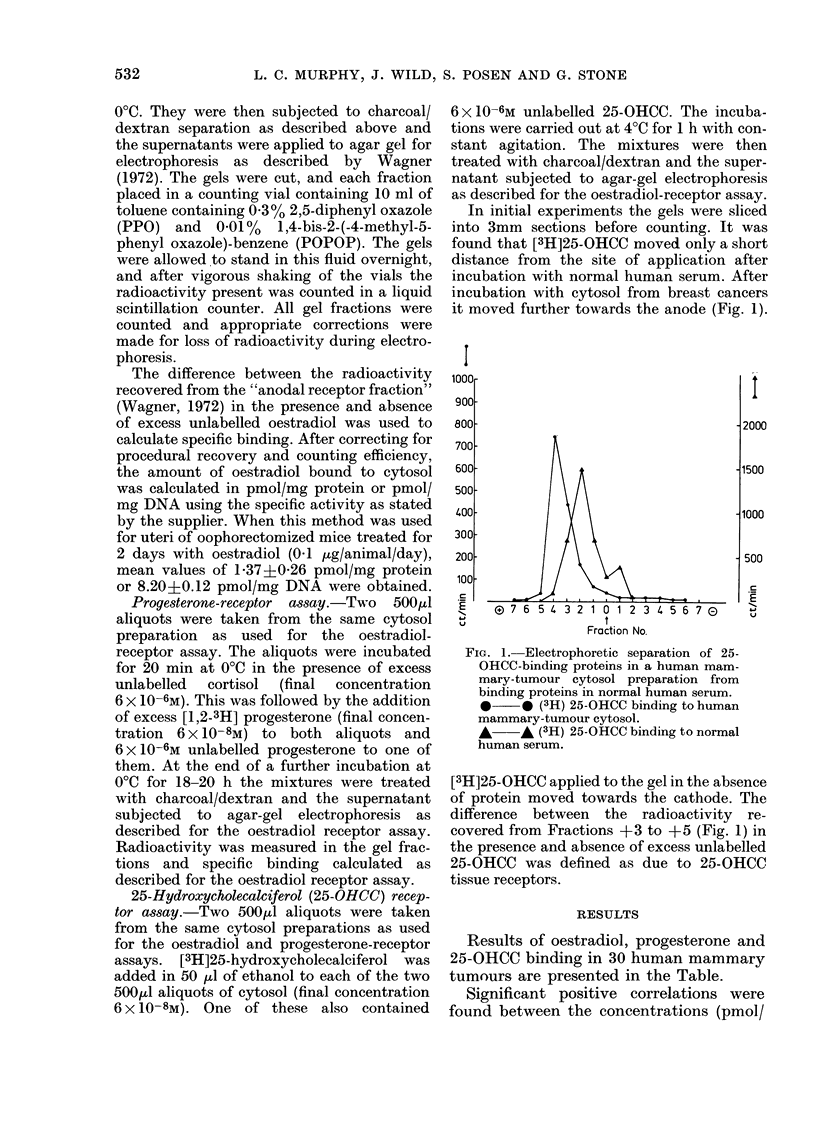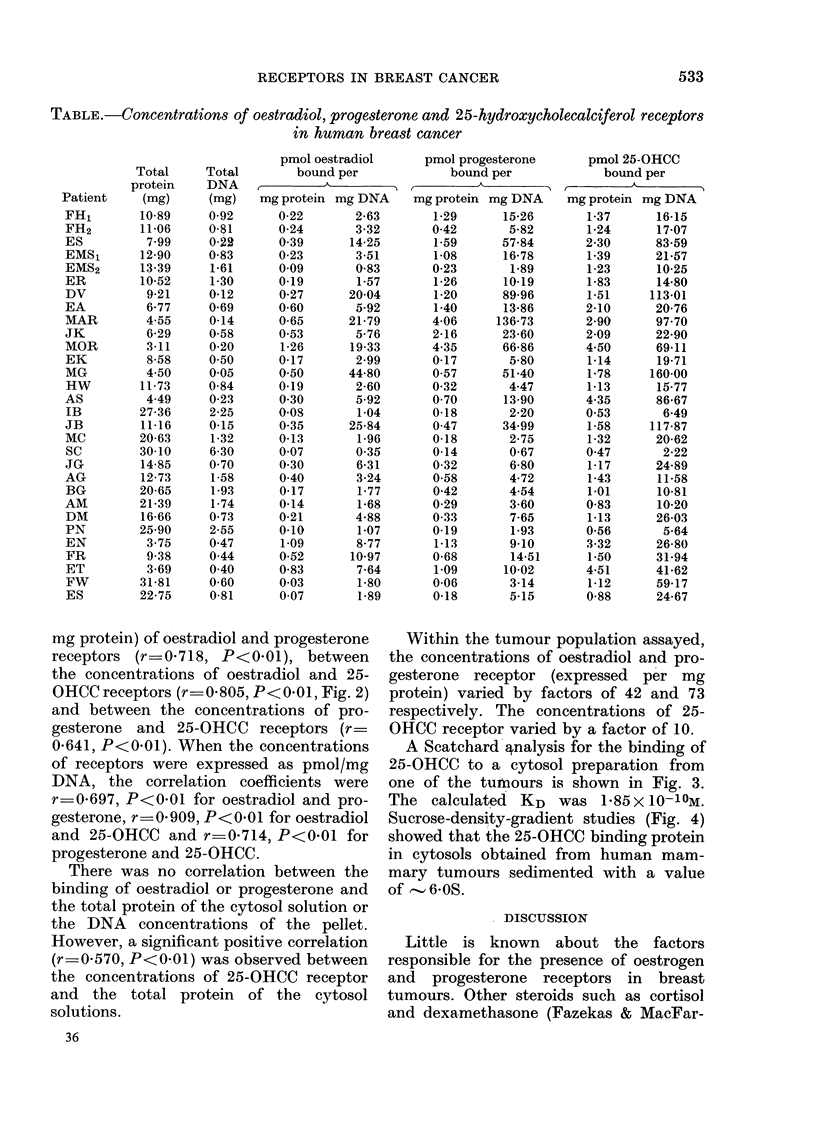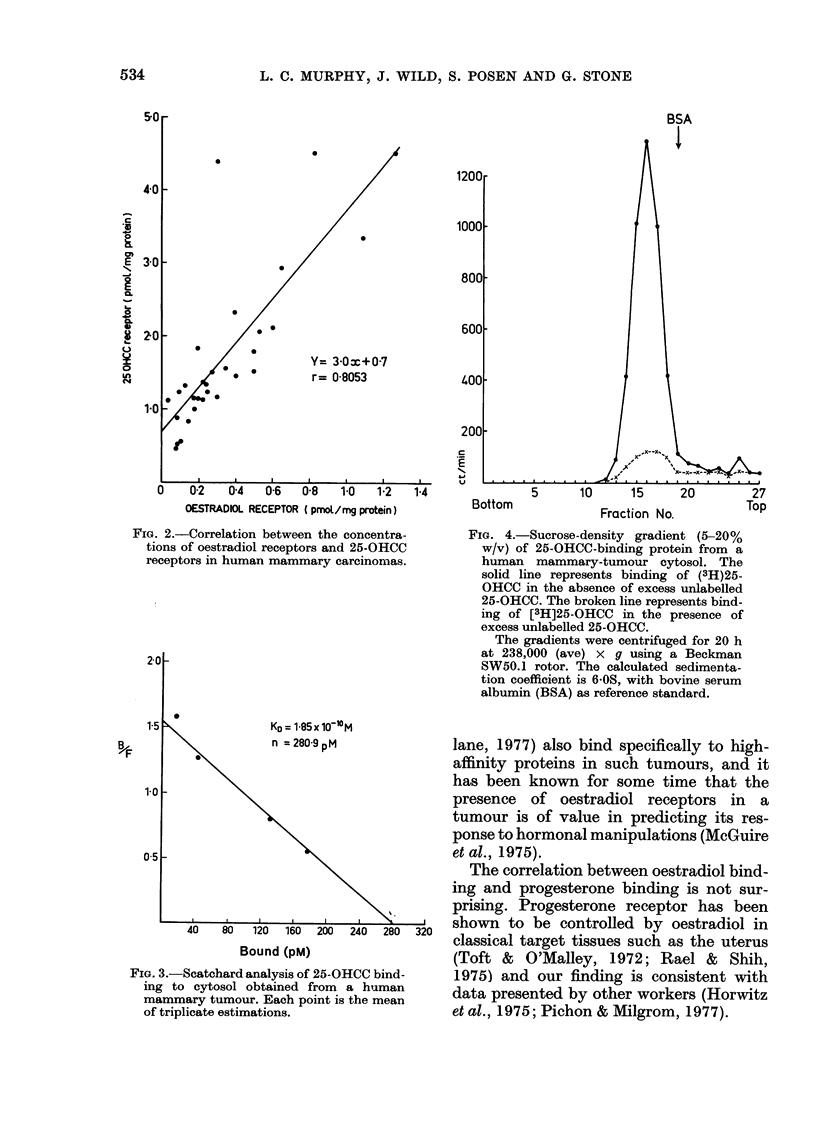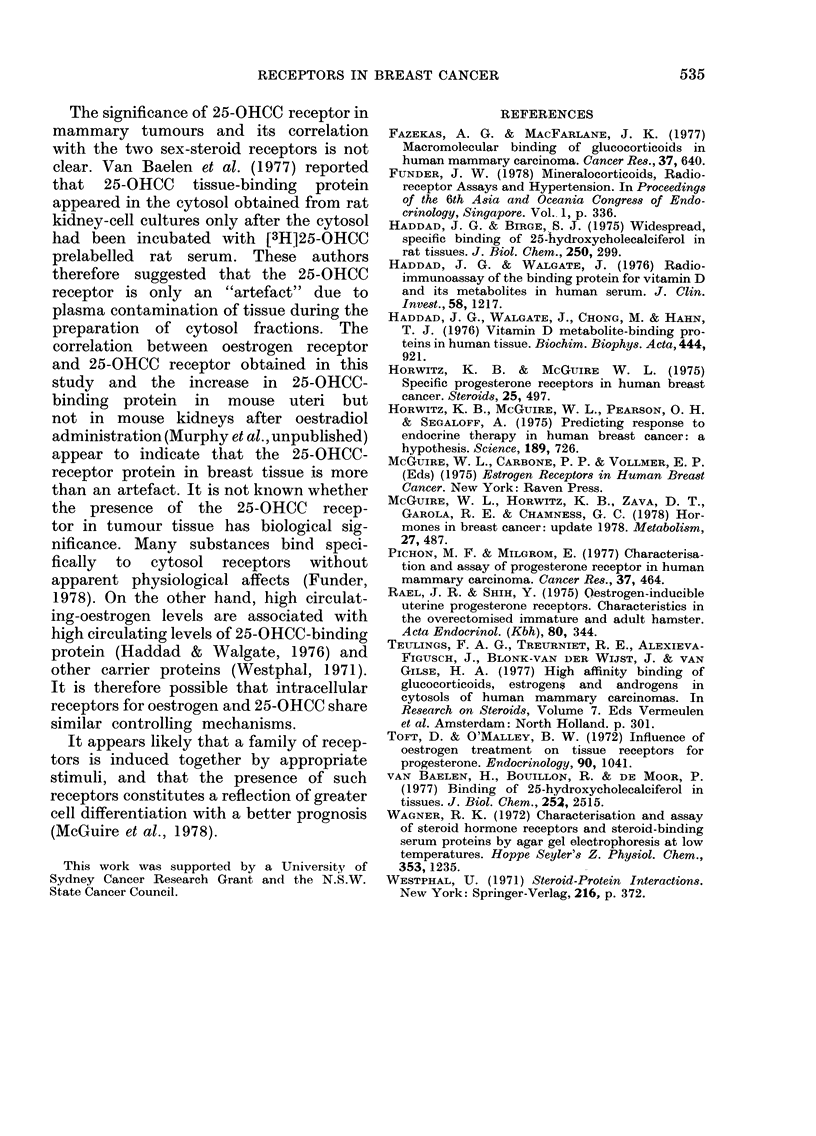# 25-Hydroxycholecalciferol receptors in human breast cancer.

**DOI:** 10.1038/bjc.1979.97

**Published:** 1979-05

**Authors:** L. C. Murphy, J. Wild, S. Posen, G. Stone

## Abstract

Cytosol receptors for 25-hydroxycholecalciferol, oestradiol and progesterone were measured in human mammary carcinomas. Significant positive correlations were found between the concentrations of all three receptors.


*Br. J. Cancer* (1979) **39**, 531

### 25-HYDROXYCHOLECALCIFEROL RECEPTORS IN HUMAN BREAST CANCER

L. C. MURPHY*, J. WILD†, S. POSEN* AND G. STONE†

*From the *Department of Medicine and the †Department of Veterinary Physiology, Sydney University, Sydney, N.S.W., Australia*

Received 25 October 1978 Accepted 8 January 1979

**Summary.—Cytosol receptors for 25-hydroxycholecalciferol, oestradiol and progesterone were measured in human mammary carcinomas. Significant positive correlations were found between the concentrations of all three receptors.**

TISSUE RECEPTORS for a number of steroids are present in human breast cancers (McGuire *et al.*, 1975; Horwitz & McGuire, 1975; Fazekas & MacFarlane, 1977; Teulings *et al.*, 1977). Binding proteins for 25-hydroxycholecalciferol (25-OHCC) have been described for all mammalian nucleated tissues so far examined (Haddad & Birge, 1975; Haddad *et al.*, 1976). We examined a number of breast tumours for the presence of 25-OHCC-binding proteins and correlated their presence with the presence of receptors for other steroids.

### MATERIALS AND METHODS

*Standard and labelled hormones.*—25-Hydroxy [26,27-$^3$H] cholecalciferol (110 Ci/mmol), [6,7-$^3$H] oestradiol-17$\beta$ (42 Ci/mmol) and [1$\alpha$,2$\alpha$-$^3$H] progesterone (49 Ci/mmol) were obtained from the Radiochemical Centre, Amersham, U.K.

Non-radioactive oestradiol-17$\beta$, progesterone and cortisol were obtained from Sigma Chemical Co., and non-radioactive 25-OHCC was a gift from Roussel Pharmaceuticals Pty. Ltd., Australia.

*Collection and processing of tissues.*—Human primary mammary carcinomas and metastases, obtained during mastectomy or by biopsy, were immediately placed on ice and obvious adipose tissue was removed. The specimens were stored in liquid $N_2$ (−196°C) and thawed just before assay.

*Preparation of cytosol solutions.*—All steps were carried out at 0–4°C unless otherwise stated. Finely minced tissue was placed in a Teflon container, immersed for several minutes in liquid $N_2$ and pulverized in a microdismembrator (Braun, Melsungen, German Federal Republic). The powder was transferred to glass centrifuge tubes and suspended in 14 volumes of buffer (0·01M Tris, 1·5mM EDTA, 0·25M sucrose, 3mM $MgCl_2$, 10% glycerol v/v, 1mM dithioerythritol) using a polypropylene micropipette tip connected to a syringe.

The suspension was centrifuged at 27,000 *g* for 20 min and the pellet retained for the estimation of DNA (Burton, 1956). The supernatant was centrifuged for 60 min at 230,000 *g* in a Beckman L2-65B ultracentrifuge and an aliquot of the supernatant (referred to as "cytosol") was assayed for protein by the Lowry method. The remainder of the cytosol solution was stirred for 10 min with a suspension containing charcoal (2·75% w/v) and dextran T70 (0·275% w/v) at a ratio of cytosol:charcoal suspension of 10:1. The mixture was centrifuged at 2000 *g* for 10 min, the supernatant was decanted and recentrifuged, and the second supernatant was used for the subsequent steps.

*Oestradiol-receptor assay.*—Two 500$\mu$l aliquots of the charcoal-treated supernatant were incubated for 20 min at 30°C with [6,7-$^3$H] oestradiol (final concentration $6\times10^{-8}$M). One of these contained, in addition, $6\times10^{-6}$M unlabelled oestradiol. The incubations were continued overnight at

Correspondence to Solomon Posen, Department of Medicine, Sydney Hospital, Sydney, N.S.W. 2001, Australia.

0°C. They were then subjected to charcoal/dextran separation as described above and the supernatants were applied to agar gel for electrophoresis as described by Wagner (1972). The gels were cut, and each fraction placed in a counting vial containing 10 ml of toluene containing 0·3% 2,5-diphenyl oxazole (PPO) and 0·01% 1,4-bis-2-(-4-methyl-5-phenyl oxazole)-benzene (POPOP). The gels were allowed to stand in this fluid overnight, and after vigorous shaking of the vials the radioactivity present was counted in a liquid scintillation counter. All gel fractions were counted and appropriate corrections were made for loss of radioactivity during electrophoresis.

The difference between the radioactivity recovered from the "anodal receptor fraction" (Wagner, 1972) in the presence and absence of excess unlabelled oestradiol was used to calculate specific binding. After correcting for procedural recovery and counting efficiency, the amount of oestradiol bound to cytosol was calculated in pmol/mg protein or pmol/mg DNA using the specific activity as stated by the supplier. When this method was used for uteri of oophorectomized mice treated for 2 days with oestradiol (0·1 μg/animal/day), mean values of $1{\cdot}37 \pm 0{\cdot}26$ pmol/mg protein or $8.20 \pm 0.12$ pmol/mg DNA were obtained.

*Progesterone-receptor assay.*—Two 500μl aliquots were taken from the same cytosol preparation as used for the oestradiol-receptor assay. The aliquots were incubated for 20 min at 0°C in the presence of excess unlabelled cortisol (final concentration $6 \times 10^{-6}$M). This was followed by the addition of excess [1,2-$^3$H] progesterone (final concentration $6 \times 10^{-8}$M) to both aliquots and $6 \times 10^{-6}$M unlabelled progesterone to one of them. At the end of a further incubation at 0°C for 18–20 h the mixtures were treated with charcoal/dextran and the supernatant subjected to agar-gel electrophoresis as described for the oestradiol receptor assay. Radioactivity was measured in the gel fractions and specific binding calculated as described for the oestradiol receptor assay.

*25-Hydroxycholecalciferol (25-OHCC) receptor assay.*—Two 500μl aliquots were taken from the same cytosol preparations as used for the oestradiol and progesterone-receptor assays. [$^3$H]25-hydroxycholecalciferol was added in 50 μl of ethanol to each of the two 500μl aliquots of cytosol (final concentration $6 \times 10^{-8}$M). One of these also contained $6 \times 10^{-6}$M unlabelled 25-OHCC. The incubations were carried out at 4°C for 1 h with constant agitation. The mixtures were then treated with charcoal/dextran and the supernatant subjected to agar-gel electrophoresis as described for the oestradiol-receptor assay.

In initial experiments the gels were sliced into 3mm sections before counting. It was found that [$^3$H]25-OHCC moved only a short distance from the site of application after incubation with normal human serum. After incubation with cytosol from breast cancers it moved further towards the anode (Fig. 1).

FIG. 1.—Electrophoretic separation of 25-OHCC-binding proteins in a human mammary-tumour cytosol preparation from binding proteins in normal human serum.
●——● ($^3$H) 25-OHCC binding to human mammary-tumour cytosol.
▲——▲ ($^3$H) 25-OHCC binding to normal human serum.

[$^3$H]25-OHCC applied to the gel in the absence of protein moved towards the cathode. The difference between the radioactivity recovered from Fractions +3 to +5 (Fig. 1) in the presence and absence of excess unlabelled 25-OHCC was defined as due to 25-OHCC tissue receptors.

### RESULTS

Results of oestradiol, progesterone and 25-OHCC binding in 30 human mammary tumours are presented in the Table.

Significant positive correlations were found between the concentrations (pmol/

TABLE.—*Concentrations of oestradiol, progesterone and 25-hydroxycholecalciferol receptors in human breast cancer*

| Patient | Total protein (mg) | Total DNA (mg) | pmol oestradiol bound per mg protein | pmol oestradiol bound per mg DNA | pmol progesterone bound per mg protein | pmol progesterone bound per mg DNA | pmol 25-OHCC bound per mg protein | pmol 25-OHCC bound per mg DNA |
|---|---|---|---|---|---|---|---|---|
| $FH_1$ | 10·89 | 0·92 | 0·22 | 2·63 | 1·29 | 15·26 | 1·37 | 16·15 |
| $FH_2$ | 11·06 | 0·81 | 0·24 | 3·32 | 0·42 | 5·82 | 1·24 | 17·07 |
| ES | 7·99 | 0·22 | 0·39 | 14·25 | 1·59 | 57·84 | 2·30 | 83·59 |
| $EMS_1$ | 12·90 | 0·83 | 0·23 | 3·51 | 1·08 | 16·78 | 1·39 | 21·57 |
| $EMS_2$ | 13·39 | 1·61 | 0·09 | 0·83 | 0·23 | 1·89 | 1·23 | 10·25 |
| ER | 10·52 | 1·30 | 0·19 | 1·57 | 1·26 | 10·19 | 1·83 | 14·80 |
| DV | 9·21 | 0·12 | 0·27 | 20·04 | 1·20 | 89·96 | 1·51 | 113·01 |
| EA | 6·77 | 0·69 | 0·60 | 5·92 | 1·40 | 13·86 | 2·10 | 20·76 |
| MAR | 4·55 | 0·14 | 0·65 | 21·79 | 4·06 | 136·73 | 2·90 | 97·70 |
| JK | 6·29 | 0·58 | 0·53 | 5·76 | 2·16 | 23·60 | 2·09 | 22·90 |
| MOR | 3·11 | 0·20 | 1·26 | 19·33 | 4·35 | 66·86 | 4·50 | 69·11 |
| EK | 8·58 | 0·50 | 0·17 | 2·99 | 0·17 | 5·80 | 1·14 | 19·71 |
| MG | 4·50 | 0·05 | 0·50 | 44·80 | 0·57 | 51·40 | 1·78 | 160·00 |
| HW | 11·73 | 0·84 | 0·19 | 2·60 | 0·32 | 4·47 | 1·13 | 15·77 |
| AS | 4·49 | 0·23 | 0·30 | 5·92 | 0·70 | 13·90 | 4·35 | 86·67 |
| IB | 27·36 | 2·25 | 0·08 | 1·04 | 0·18 | 2·20 | 0·53 | 6·49 |
| JB | 11·16 | 0·15 | 0·35 | 25·84 | 0·47 | 34·99 | 1·58 | 117·87 |
| MC | 20·63 | 1·32 | 0·13 | 1·96 | 0·18 | 2·75 | 1·32 | 20·62 |
| SC | 30·10 | 6·30 | 0·07 | 0·35 | 0·14 | 0·67 | 0·47 | 2·22 |
| JG | 14·85 | 0·70 | 0·30 | 6·31 | 0·32 | 6·80 | 1·17 | 24·89 |
| AG | 12·73 | 1·58 | 0·40 | 3·24 | 0·58 | 4·72 | 1·43 | 11·58 |
| BG | 20·65 | 1·93 | 0·17 | 1·77 | 0·42 | 4·54 | 1·01 | 10·81 |
| AM | 21·39 | 1·74 | 0·14 | 1·68 | 0·29 | 3·60 | 0·83 | 10·20 |
| DM | 16·66 | 0·73 | 0·21 | 4·88 | 0·33 | 7·65 | 1·13 | 26·03 |
| PN | 25·90 | 2·55 | 0·10 | 1·07 | 0·19 | 1·93 | 0·56 | 5·64 |
| EN | 3·75 | 0·47 | 1·09 | 8·77 | 1·13 | 9·10 | 3·32 | 26·80 |
| FR | 9·38 | 0·44 | 0·52 | 10·97 | 0·68 | 14·51 | 1·50 | 31·94 |
| ET | 3·69 | 0·40 | 0·83 | 7·64 | 1·09 | 10·02 | 4·51 | 41·62 |
| FW | 31·81 | 0·60 | 0·03 | 1·80 | 0·06 | 3·14 | 1·12 | 59·17 |
| ES | 22·75 | 0·81 | 0·07 | 1·89 | 0·18 | 5·15 | 0·88 | 24·67 |

mg protein) of oestradiol and progesterone receptors ($r=0{\cdot}718$, $P<0{\cdot}01$), between the concentrations of oestradiol and 25-OHCC receptors ($r=0{\cdot}805$, $P<0{\cdot}01$, Fig. 2) and between the concentrations of progesterone and 25-OHCC receptors ($r=0{\cdot}641$, $P<0{\cdot}01$). When the concentrations of receptors were expressed as pmol/mg DNA, the correlation coefficients were $r=0{\cdot}697$, $P<0{\cdot}01$ for oestradiol and progesterone, $r=0{\cdot}909$, $P<0{\cdot}01$ for oestradiol and 25-OHCC and $r=0{\cdot}714$, $P<0{\cdot}01$ for progesterone and 25-OHCC.

There was no correlation between the binding of oestradiol or progesterone and the total protein of the cytosol solution or the DNA concentrations of the pellet. However, a significant positive correlation ($r=0{\cdot}570$, $P<0{\cdot}01$) was observed between the concentrations of 25-OHCC receptor and the total protein of the cytosol solutions.

Within the tumour population assayed, the concentrations of oestradiol and progesterone receptor (expressed per mg protein) varied by factors of 42 and 73 respectively. The concentrations of 25-OHCC receptor varied by a factor of 10.

A Scatchard analysis for the binding of 25-OHCC to a cytosol preparation from one of the tumours is shown in Fig. 3. The calculated $K_D$ was $1{\cdot}85\times10^{-10}$M. Sucrose-density-gradient studies (Fig. 4) showed that the 25-OHCC binding protein in cytosols obtained from human mammary tumours sedimented with a value of $\sim 6{\cdot}0$S.

### DISCUSSION

Little is known about the factors responsible for the presence of oestrogen and progesterone receptors in breast tumours. Other steroids such as cortisol and dexamethasone (Fazekas & MacFar-

FIG. 2.—Correlation between the concentrations of oestradiol receptors and 25-OHCC receptors in human mammary carcinomas.

FIG. 3.—Scatchard analysis of 25-OHCC binding to cytosol obtained from a human mammary tumour. Each point is the mean of triplicate estimations.

FIG. 4.—Sucrose-density gradient (5–20% w/v) of 25-OHCC-binding protein from a human mammary-tumour cytosol. The solid line represents binding of ($^3$H)25-OHCC in the absence of excess unlabelled 25-OHCC. The broken line represents binding of [$^3$H]25-OHCC in the presence of excess unlabelled 25-OHCC.

The gradients were centrifuged for 20 h at 238,000 (ave) × *g* using a Beckman SW50.1 rotor. The calculated sedimentation coefficient is 6·0S, with bovine serum albumin (BSA) as reference standard.

lane, 1977) also bind specifically to high-affinity proteins in such tumours, and it has been known for some time that the presence of oestradiol receptors in a tumour is of value in predicting its response to hormonal manipulations (McGuire *et al.*, 1975).

The correlation between oestradiol binding and progesterone binding is not surprising. Progesterone receptor has been shown to be controlled by oestradiol in classical target tissues such as the uterus (Toft & O'Malley, 1972; Rael & Shih, 1975) and our finding is consistent with data presented by other workers (Horwitz *et al.*, 1975; Pichon & Milgrom, 1977).

The significance of 25-OHCC receptor in mammary tumours and its correlation with the two sex-steroid receptors is not clear. Van Baelen *et al.* (1977) reported that 25-OHCC tissue-binding protein appeared in the cytosol obtained from rat kidney-cell cultures only after the cytosol had been incubated with [$^3$H]25-OHCC prelabelled rat serum. These authors therefore suggested that the 25-OHCC receptor is only an "artefact" due to plasma contamination of tissue during the preparation of cytosol fractions. The correlation between oestrogen receptor and 25-OHCC receptor obtained in this study and the increase in 25-OHCC-binding protein in mouse uteri but not in mouse kidneys after oestradiol administration (Murphy *et al.*, unpublished) appear to indicate that the 25-OHCC-receptor protein in breast tissue is more than an artefact. It is not known whether the presence of the 25-OHCC receptor in tumour tissue has biological significance. Many substances bind specifically to cytosol receptors without apparent physiological affects (Funder, 1978). On the other hand, high circulating-oestrogen levels are associated with high circulating levels of 25-OHCC-binding protein (Haddad & Walgate, 1976) and other carrier proteins (Westphal, 1971). It is therefore possible that intracellular receptors for oestrogen and 25-OHCC share similar controlling mechanisms.

It appears likely that a family of receptors is induced together by appropriate stimuli, and that the presence of such receptors constitutes a reflection of greater cell differentiation with a better prognosis (McGuire *et al.*, 1978).

This work was supported by a University of Sydney Cancer Research Grant and the N.S.W. State Cancer Council.

### REFERENCES

FAZEKAS, A. G. & MACFARLANE, J. K. (1977) Macromolecular binding of glucocorticoids in human mammary carcinoma. *Cancer Res.*, **37**, 640.

FUNDER, J. W. (1978) Mineralocorticoids, Radioreceptor Assays and Hypertension. In *Proceedings of the 6th Asia and Oceania Congress of Endocrinology, Singapore*. Vol. 1, p. 336.

HADDAD, J. G. & BIRGE, S. J. (1975) Widespread, specific binding of 25-hydroxycholecalciferol in rat tissues. *J. Biol. Chem.*, **250**, 299.

HADDAD, J. G. & WALGATE, J. (1976) Radioimmunoassay of the binding protein for vitamin D and its metabolites in human serum. *J. Clin. Invest.*, **58**, 1217.

HADDAD, J. G., WALGATE, J., CHONG, M. & HAHN, T. J. (1976) Vitamin D metabolite-binding proteins in human tissue. *Biochim. Biophys. Acta*, **444**, 921.

HORWITZ, K. B. & MCGUIRE W. L. (1975) Specific progesterone receptors in human breast cancer. *Steroids*, **25**, 497.

HORWITZ, K. B., MCGUIRE, W. L., PEARSON, O. H. & SEGALOFF, A. (1975) Predicting response to endocrine therapy in human breast cancer: a hypothesis. *Science*, **189**, 726.

MCGUIRE, W. L., CARBONE, P. P. & VOLLMER, E. P. (Eds) (1975) *Estrogen Receptors in Human Breast Cancer*. New York: Raven Press.

MCGUIRE, W. L., HORWITZ, K. B., ZAVA, D. T., GAROLA, R. E. & CHAMNESS, G. C. (1978) Hormones in breast cancer: update 1978. *Metabolism*, **27**, 487.

PICHON, M. F. & MILGROM, E. (1977) Characterisation and assay of progesterone receptor in human mammary carcinoma. *Cancer Res.*, **37**, 464.

RAEL, J. R. & SHIH, Y. (1975) Oestrogen-inducible uterine progesterone receptors. Characteristics in the overectomised immature and adult hamster. *Acta Endocrinol.* (*Kbh*), **80**, 344.

TEULINGS, F. A. G., TREURNIET, R. E., ALEXIEVA-FIGUSCH, J., BLONK-VAN DER WIJST, J. & VAN GILSE, H. A. (1977) High affinity binding of glucocorticoids, estrogens and androgens in cytosols of human mammary carcinomas. In *Research on Steroids*, Volume 7. Eds Vermeulen *et al.* Amsterdam: North Holland. p. 301.

TOFT, D. & O'MALLEY, B. W. (1972) Influence of oestrogen treatment on tissue receptors for progesterone. *Endocrinology*, **90**, 1041.

VAN BAELEN, H., BOUILLON, R. & DE MOOR, P. (1977) Binding of 25-hydroxycholecalciferol in tissues. *J. Biol. Chem.*, **252**, 2515.

WAGNER, R. K. (1972) Characterisation and assay of steroid hormone receptors and steroid-binding serum proteins by agar gel electrophoresis at low temperatures. *Hoppe Seyler's Z. Physiol. Chem.*, **353**, 1235.

WESTPHAL, U. (1971) *Steroid-Protein Interactions*. New York: Springer-Verlag, **216**, p. 372.